# No Correlation Between Proteinuria and Renal Function in Patients with Unresectable Hepatocellular Carcinoma Treated with Atezolizumab Plus Bevacizumab: ARISE Study †

**DOI:** 10.3390/cancers17233826

**Published:** 2025-11-28

**Authors:** Kazuomi Ueshima, Naoshi Nishida, Satoru Hagiwara, Yasunori Minami, Hiroshi Ida, Masahiro Takita, Hirokazu Chishina, Masahiro Morita, Tomoko Aoki, Tetsutaro Hamano, Ryosuke Take, Chizuko Watanabe, Kohsuke Asoh, Ai Tanaka, Masatoshi Kudo

**Affiliations:** 1 Department of Gastroenterology and Hepatology, Kindai University Faculty of Medicine, Osaka 589-8511, Japan; naoshi@med.kindai.ac.jp (N.N.); hagi-318@hotmail.co.jp (S.H.); minkun@med.kindai.ac.jp (Y.M.); hidakuhp@gmail.com (H.I.); masahirot2797@yahoo.co.jp (M.T.); chocolatecake3candy@yahoo.co.jp (H.C.); s0750081@yahoo.co.jp (M.M.); tomoko.aoki@med.kindai.ac.jp (T.A.); m-kudo@med.kindai.ac.jp (M.K.); 2 P4 Statistics Co., Ltd., Tokyo 158-0082, Japan; hamano@p4st.jp; 3 Department of Oncology Medical Science, Chugai Pharmaceutical Co., Ltd., Tokyo 103-8324, Japan

**Keywords:** atezolizumab, bevacizumab, unresectable hepatocellular carcinoma, proteinuria, renal function

## Abstract

**Background:** Atezolizumab plus bevacizumab (Atezo + Bev) is the standard of care for treatment-naïve patients with unresectable hepatocellular carcinoma (uHCC). Proteinuria is a treatment-emergent adverse event that often leads to Bev interruption. However, the relationship between Bev-related proteinuria and renal dysfunction is unclear. We retrospectively investigated the impact of proteinuria after starting Atezo + Bev on renal function in patients with uHCC. **Methods:** We performed a single-arm retrospective study of patients with uHCC treated with Atezo + Bev between 25 September 2020 and 31 May 2022, at Kindai University Hospital, Japan. The impact of proteinuria on renal function during Atezo + Bev treatment was analyzed in terms of the correlation between changes in urine protein creatinine ratio (UPCR) and estimated glomerular filtration rate (eGFR) relative to baseline. **Results:** We analyzed data from 100 patients (median age 74 years; range 41–89; 75% male). During Atezo + Bev treatment, the median (interquartile range) maximum increase from baseline in UPCR was 0.39 (0.08 to 2.05) and the median maximum decline from baseline in eGFR was −7.5 (−20.5 to −3.0) mL/min/1.73 m^2^. The Pearson and Spearman correlation coefficients (95% confidence intervals) between these variables were −0.16 (−0.34 to 0.04) and −0.13 (−0.32 to 0.07), respectively. **Conclusions:** We found no correlation between the changes in UPCR and eGFR during Atezo + Bev treatment. Bev interruption criteria are based on the degree of proteinuria; however, our results suggest that proteinuria does not necessarily impair renal function. Physicians should consider the risk–benefit profile when deciding whether to discontinue Bev in patients who develop proteinuria during Atezo + Bev treatment.

## Simple Summary

Patients with unresectable hepatocellular carcinoma are often treated with atezolizumab and bevacizumab (Atezo + Bev). Proteinuria, a treatment-emergent adverse event caused by Bev, may force the suspension or discontinuation of Bev for safety reasons. In this study, we found that the urine protein level was not related to the deterioration of renal function in patients treated with Atezo + Bev. Accordingly, physicians should consider the risk–benefit profile when deciding whether to stop Bev in patients with above-normal urine protein levels during treatment with Atezo + Bev.

## 1. Introduction

The combination of the anti-programmed cell death ligand-1 (PD-L1) and anti-vascular endothelial growth factor (VEGF) antibodies atezolizumab and bevacizumab (Atezo + Bev) is a standard of care for patients with untreated unresectable hepatocellular carcinoma (uHCC) based on results of the IMbrave150 trial [1,2].

Since then, the first-line treatment landscape has expanded. Dual immune checkpoint inhibitor (ICI) combinations have demonstrated prolonged survival compared to tyrosine kinase inhibitors, including durvalumab plus tremelimumab based on the HIMALAYA trial and nivolumab plus ipilimumab based on the CheckMate 9DW trial [3,4,5]. Furthermore, the role of systemic therapy has expanded to include patients with intermediate-stage HCC, particularly as a first-line option for patients considered unsuitable for transcatheter arterial chemoembolization (TACE) [6,7,8]. Notably, several recent international phase 3 trials have demonstrated that the combination of TACE with an ICI and an anti-VEGF agent significantly prolongs progression-free survival compared to TACE alone [9,10].

Despite the emergence of dual ICI regimens, an ICI combined with an anti-VEGF agent, such as Atezo + Bev, remains an important treatment option for uHCC. Optimizing the management of these regimens is crucial to maximize patient outcomes. An essential aspect involves managing treatment-related adverse events (AEs). Because Bev is known to enhance the efficacy of Atezo, continuing it for as long as possible by effectively managing its AEs is essential [11].

Proteinuria is a major AE that leads to Bev interruption and is considered to be a risk factor for the progression of chronic kidney disease and renal failure [12]. This can be particularly problematic in patients with HCC because persistent proteinuria may lead to a decrease in the serum albumin level in people whose albumin synthesis capacity is often already reduced. Some studies have suggested that proteinuria and low serum albumin could be reversed after discontinuation of the anti-VEGF agent [13,14].

According to the Clinical Practice Guidelines for the Management of Kidney Injury During Anticancer Drug Therapy 2022 [15,16], if proteinuria develops during anti-VEGF therapy, the decision to continue treatment should be based on the risk–benefit assessment and the patient’s preference, given the limited prognosis of patients with advanced cancer. Careful management of proteinuria is crucial, but interrupting or discontinuing treatment could negatively affect the patient’s prognosis [17,18].

To date, however, the association between proteinuria and safety outcomes, including renal dysfunction and hypoalbuminemia, has not been adequately investigated in patients with uHCC. Therefore, the aim of this retrospective study was to evaluate the association between proteinuria and renal function, as well as serum albumin levels, after initiating Atezo + Bev in patients with uHCC.

## 2. Materials and Methods

### 2.1. Study Design and Patients

This was a single-center, retrospective study performed at Kindai University Hospital in Japan (registration number: UMIN000050692). We retrieved and analyzed the medical records of adult patients with uHCC who started Atezo + Bev between 25 September 2020 and 31 May 2022, and were ≥18 years old at the start of Atezo + Bev treatment. Patients were excluded from the study for the following reasons: absence of urine protein creatinine ratio (UPCR) and/or estimated glomerular filtration rate (eGFR) data at baseline or after starting Atezo + Bev, and patients judged to be ineligible by the investigator. All patients underwent esophagogastroduodenoscopy (EGD) within 6 months prior to treatment initiation, and any high-risk esophageal varices were treated prophylactically. Patients with varices not requiring prophylactic treatment at baseline underwent follow-up EGD every 3–6 months. For patients with Barcelona Clinic Liver Cancer (BCLC) stage B, Atezo + Bev was administered to those who were considered unsuitable for or refractory to TACE, in line with expert consensus and clinical guidelines [19,20,21]. According to clinical guidelines [15,16], the study population included patients who continued Atezo + Bev treatment despite developing proteinuria with UPCR ≥ 2.0 after careful evaluation of potential risks and benefits in individual cases.

### 2.2. Endpoints and Data Collection

The primary endpoint of this study was to determine the correlation between the change in UPCR and the change in eGFR from baseline during Atezo + Bev treatment. The secondary and exploratory endpoints were to determine the correlation between UPCR and urine protein dipstick test results, the correlation between proteinuria and hypoalbuminemia, and risk factors for renal function decline and hypoalbuminemia. To investigate these endpoints, we retrieved the following data from the patients’ medical records: baseline data (within 28 days before or at the start of Atezo + Bev treatment) (age, sex, blood pressure, Eastern Cooperative Oncology Group performance status [ECOG PS], medical history and comorbidities, liver disease, ascites/encephalopathy, vascular invasion, extrahepatic metastasis, treatment line for Atezo + Bev, treatment history, date of starting Atezo + Bev, blood biochemistry, dipstick urine protein test, and prothrombin time–international normalized ratio) and data during treatment (date of Atezo + Bev administration, date and reason for interruption of Atezo and Bev, blood biochemistry, and dipstick urine protein test results).

### 2.3. Statistical Analyses

As a retrospective analysis, the sample size was not planned a priori, but we estimated that approximately 100 patients would be eligible for the study.

The primary endpoint (the correlation between the change in UPCR from baseline and the change in eGFR from baseline) was determined as follows. UPCR and eGFR were measured for each treatment cycle, and the changes were calculated relative to baseline. Based on the hypothesis that worse proteinuria implies lower eGFR, we calculated the maximum increase in log_2_-transformed UPCR from baseline and the maximum decline in eGFR from baseline during the Atezo + Bev treatment period for each patient. We used Pearson’s correlation coefficient and Spearman’s rank correlation coefficient to determine correlations between the change in UPCR and the change in eGFR in all patients and in patients divided according to the treatment line (1st-line, 2nd/later-line). We also determined the correlation between the mean change in log_2_-transformed UPCR and the mean change in eGFR.

The secondary and exploratory endpoints were as follows. Based on the hypothesis that higher UPCR implies lower serum albumin, we calculated the maximum increase in log_2_-transformed UPCR from baseline and the maximum decrease in serum albumin from baseline during the Atezo + Bev treatment period for each patient. We then used Pearson’s correlation coefficient and Spearman’s rank coefficient to determine the correlations between the changes in UPCR and serum albumin. Univariate and multivariable logistic regression was performed to identify factors associated with (1) a decline in renal function and (2) the occurrence of hypoalbuminemia during treatment with Atezo + Bev. In the first analysis, a decline in renal function was defined as a decline in eGFR to <50 mL/min/1.73 m^2^. The analysis excluded patients with eGFR < 50 mL/min/1.73 m^2^ at baseline. In the second analysis, hypoalbuminemia was defined as a serum albumin level of <3.0 g/dL. The analysis excluded patients with serum albumin < 3.0 g/dL at baseline. Both models included the covariate age, sex, comorbidities, BCLC stage, Child–Pugh class, treatment line, history of TACE, and baseline laboratory variables (UPCR, eGFR, serum albumin, and blood urea nitrogen [BUN]). Comorbidities included in the analysis were diabetes, hypertension (systolic blood pressure ≥ 140 mmHg), and chronic kidney failure. Clinical laboratory variables were divided into high and low values according to the following cutoff values. For UPCR, the value 0.15 was used because this is a reference value for positive proteinuria. An eGFR of 60 mL/min/1.73 m^2^ is indicative of chronic kidney disease with mild renal function decline and is defined as Grade 2 in the Common Terminology Criteria for Adverse Events. The cutoff for serum albumin was set at 3.5 g/dL, which corresponds to the threshold value for changes in Child–Pugh grades. For BUN, we used the median value. A stepwise method was used to select the final variables included in the multivariable model. Odds ratios (ORs) were calculated with Wald confidence intervals (CIs), with a null hypothesis of an OR of 1.

In correlation and regression analyses, we used log_2_-transformed UPCR to approximate a normal distribution.

To explore the potential association between the on-treatment renal function decline and overall survival (OS), we performed an exploratory analysis in a subgroup of patients with normal baseline renal function (defined as eGFR ≥ 60 mL/min/1.73 m^2^). Patients were categorized into two groups according to their renal function during treatment: a “decline group” (patients whose eGFR declined to <50 mL/min/1.73 m^2^) and a “non-decline group” (patients whose eGFR remained ≥ 50 mL/min/1.73 m^2^).

OS was calculated from the date of Atezo + Bev initiation to the date of death from any cause. The Kaplan–Meier method was used to estimate median OS, and survival distributions were compared using the log-rank test. The Cox proportional hazards model was used to calculate the hazard ratio (HR) with 95% CIs.

Significance was accepted at a level of 5% (*p* < 0.05). All statistical analyses were performed using SAS version 9.4 (SAS Institute, Cary, NC, USA).

## 3. Results

### 3.1. Patients

Overall, 100 patients with uHCC who were treated with Atezo + Bev during the study period and satisfied the eligibility criteria were included in the analysis. The median age was 74 years, and 75% of the patients were male (Table 1). The BCLC stages were B in 62% and C in 38%. Atezo + Bev was used as 1st-line therapy in 51% of patients and as 2nd/later-line therapy in 49%. For the 49 patients receiving Atezo + Bev as 2nd/later-line therapy, prior systemic treatments included lenvatinib (*n* = 42; 86%), investigational drugs (*n* = 29; 59%), sorafenib (*n* = 17; 35%), and other therapies (*n* = 13; 27%). The median baseline UPCR and eGFR were 0.10 g/g creatinine (Cre) and 68.5 mL/min/1.73 m^2^, respectively. Urine dipstick protein was negative in 55% of patients. The median serum albumin level was 3.7 g/dL. At baseline, 10 patients (10%) had hypoalbuminemia, defined as a serum albumin level < 3.0 g/dL. In these 10 patients, the Child–Pugh class was A6 in two and B in eight.

### 3.2. Correlation Between UPCR and Urine Dipstick Test

**Figure 1:**
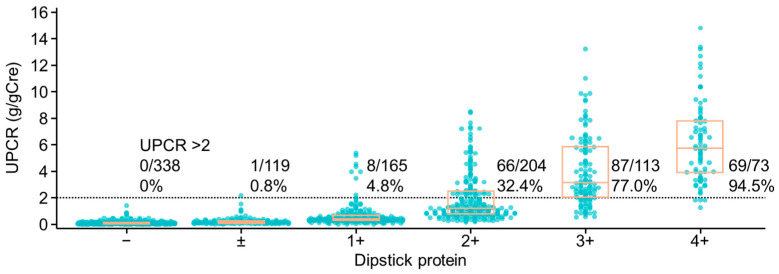
Correlation between UPCR and urine protein dipstick test.

We first examined the correlation between UPCR and urine dipstick test. However, as illustrated in Figure 1, there was a lack of concordance between patients with UPCR ≥ 2.0 g/gCre and those with positive urine dipstick test results.

### 3.3. Correlation Between Changes in UPCR and eGFR from Baseline

**Figure 2:**
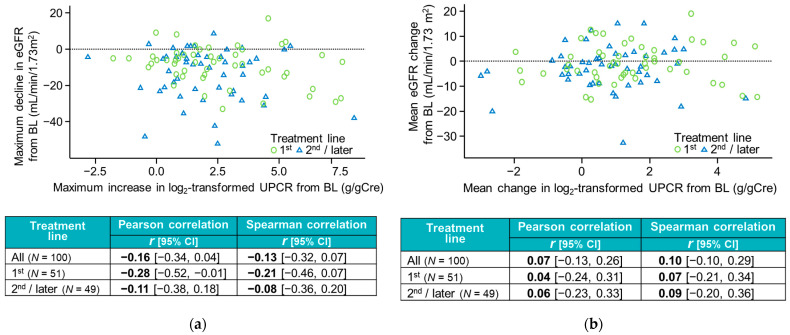
Correlations between the maximum increase in log_2_ UPCR and the maximum decline in eGFR (**a**) and between the mean change in log_2_ UPCR and mean change in eGFR from baseline (**b**) in all patients and according to treatment line. BL, baseline; CI, confidence interval.

During Atezo + Bev treatment, the median (interquartile range) maximum increase from baseline in UPCR was 0.39 (0.08 to 2.05) and the median maximum decline from baseline in eGFR was −7.5 (−20.5 to −3.0) mL/min/1.73 m^2^. As depicted in Figure 2, we found no correlation between the maximum increase in log_2_-transformed UPCR and the maximum decline in eGFR from baseline in either the overall population or the subgroups by treatment line. This lack of correlation was apparent using both the Pearson’s and Spearman’s correlation coefficients.

### 3.4. Risk Factors for the Decline in Renal Function

We next sought to identify potential risk factors for a decline in renal function during treatment with Atezo + Bev by performing univariate and multivariable logistic regression analyses, the results of which are presented in Table 2. In the univariate analyses, hypertension, low eGFR (≤60 mL/min/1.73 m^2^), and history of TACE were significantly associated with a decline in renal function to <50 mL/min/1.73 m^2^. After applying stepwise multiple logistic regression, the only significant risk factor was low eGFR (≤60 mL/min/1.73 m^2^).

### 3.5. Correlation Between Changes in UPCR and Serum Albumin Levels from Baseline

**Figure 3:**
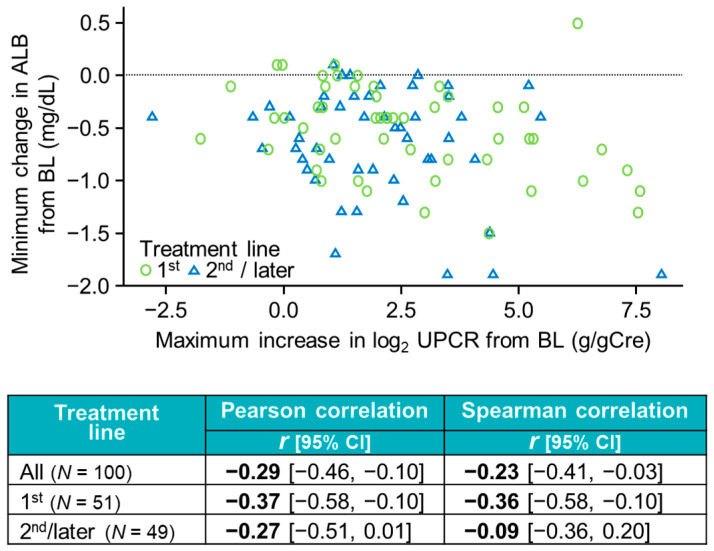
Correlation between the maximum increase in log_2_-transformed UPCR and the minimum change in serum albumin from baseline in all patients and according to treatment line. ALB, albumin.

To further examine the potential relevance of renal function during Atezo + Bev treatment, we examined the potential correlation between the maximum increase in log_2_ UPCR and the maximum decline in serum albumin from baseline. As shown in Figure 3, there were weak correlations between these variables in the overall population and in patients who received Atezo + Bev as 1st-line treatment, but not in patients who received Atezo + Bev as 2nd/later-line treatment.

### 3.6. Exploratory Analysis of the Association Between Renal Function Decline and OS

To explore the association between the on-treatment renal function decline and OS, we conducted an analysis in the subgroup of 65 patients with normal renal function (eGFR ≥ 60 mL/min/1.73 m^2^) at baseline, which was subsequently divided into the non-decline group (eGFR ≥ 50 mL/min/1.73 m^2^; *n* = 55) and the decline group (eGFR < 50 mL/min/1.73 m^2^; *n* = 10) according to the change in eGFR during treatment.

Several imbalances in baseline characteristics were observed between these two groups. Compared to the non-decline group, the decline group included a smaller proportion of males (50.0% vs. 81.8%) and greater proportions of patients with BCLC stage B (80.0% vs. 50.9%), patients receiving 2nd/later-line therapy (70.0% vs. 45.5%), and patients with hypertension (70.0% vs. 43.6%). The decline group also had a tendency toward worse baseline renal function, with a lower median eGFR (71.0 vs. 78.0 mL/min/1.73 m^2^), higher median UPCR (0.35 vs. 0.08), and a greater proportion of patients with a urine dipstick result of ≥2+ (40.0% vs. 14.5%) (Supplementary Table S1).

Although there was no significant difference in OS between the two groups (*p* = 0.387), the median OS tended to be shorter in the decline group (14.8 months, 95% CI: 3.9, not reached) than in the non-decline group (24.0 months, 95% CI: 14.6, not reached) (Supplementary Figure S1). The HR for death in the decline group compared to the non-decline group was 1.45 (95% CI: 0.62, 3.39).

### 3.7. Risk Factors for Hypoalbuminemia

In the final part of the study, we examined potential risk factors for hypoalbuminemia (serum albumin < 3.0 g/dL). Although relatively large ORs were obtained for several baseline factors in the univariate analyses, including UPCR ≥ 0.15 (3.31; *p* = 0.068), chronic kidney failure (2.50; *p* = 0.282), and serum albumin ≤ 3.5 g/dL (1.92; *p* = 0.203), none of the variables were statistically significant (Table 3). Therefore, multiple regression analysis was not performed.

## 4. Discussion

To our knowledge, this was the first study to investigate the potential relationships between proteinuria and renal function and serum albumin levels during Atezo + Bev treatment in patients with uHCC. Of note, we found no significant correlation between the change in UPCR and the change in eGFR during treatment with Atezo + Bev, although there was a weak correlation between the change in UPCR and the change in serum albumin, primarily in patients who received Atezo + Bev as 1st-line treatment.

Bev-induced proteinuria is a clinically important event that may prompt changes to anticancer treatment regimens, particularly in patients with HCC. However, the mechanisms underlying Bev-induced proteinuria remain to be fully elucidated. One hypothesis is that inhibiting VEGF, which plays a crucial role in maintaining the function of renal glomerular capillary endothelial cells, may impede the repair of glomerular capillaries and diminish glomerular filtration capacity [22]. Notably, our findings are consistent with previous reports in other cancers showing no significant correlation between proteinuria and renal function decline during anti-angiogenic therapy [23,24,25,26,27,28]. However, contrasting results were reported in other studies [29,30,31], and we cannot ignore the possibility that the differing findings may be attributed to drug-specific effects, patient characteristics, and observation periods, among other factors.

Our study cohort included a large proportion of patients with known risk factors for renal decline, such as hypertension and chronic kidney disease [32]. In a multivariable analysis of predictors for renal decline during treatment (eGFR <50 mL/min/1.73 m^2^), the strongest independent predictor was a baseline eGFR of ≤60 mL/min/1.73 m^2^ (OR 3.72), while baseline hypertension showed a trend toward increased risk (OR 2.70). The presence of these comorbidities inherently complicates the interpretation of a direct causal relationship between treatment-induced proteinuria and the decline in renal function during treatment.

Since our study population reflects real-world clinical practice in an aging Japan [33], our primary finding—the lack of a correlation between the degree of proteinuria and the extent of the renal function decline in a population with high underlying risk—has important clinical implications. This finding suggests that proteinuria during Atezo + Bev treatment is not necessarily a direct indicator of a progressive decline in renal function.

The results of our study may contradict the established concept that proteinuria is a risk factor for future renal failure [12,34]. Although the reason for this apparent discrepancy is unclear, we speculate that it is partly due to the relatively short cancer treatment period in this study compared to the long-term effects of chronic proteinuria, and because Bev-induced proteinuria improves upon discontinuation or interruption of Bev [35].

Our exploratory analysis revealed a trend toward shorter OS in patients experiencing renal decline during treatment (HR 1.45, *p* = 0.387). This finding should be interpreted with caution due to the small sample size and the potential existence of confounding baseline characteristics. Although causality cannot be definitively established, the association between renal decline during treatment and worse prognosis emphasizes the clinical necessity for ongoing eGFR monitoring to identify at-risk patients and allow timely intervention.

Intriguingly, we found a weak correlation between proteinuria and serum albumin, although the clinical significance of this finding is unclear. We believe this is the first study to examine the potential correlation between these factors during anti-angiogenic therapy. Although some studies have suggested that proteinuria may affect serum albumin and albumin-bilirubin (ALBI) scores [36], a clear causal relationship has not been established. Importantly, the IMbrave150 study found no decline in ALBI scores, suggesting that the decrease in serum albumin is not solely attributable to Bev-induced proteinuria [37]. Rather, the decrease in serum albumin levels in patients with HCC may be influenced by multiple factors, including liver function and nutritional status [38,39,40].

Finally, we searched for potential risk factors for hypoalbuminemia during Atezo + Bev therapy. However, we found no significant variables in the univariate analyses. These results suggest that the development of hypoalbuminemia during Atezo + Bev treatment may be multifactorial and is not solely dependent on proteinuria. However, it is entirely possible that hypoalbuminemia can be caused by the loss of albumin due to long-term severe proteinuria, such as in nephrotic syndrome. Therefore, proteinuria due to Bev could be a predictor of hypoalbuminemia. However, because this study assessed proteinuria and serum albumin over a short period of time, the true relationship may not have been fully evaluated.

Our results should encourage physicians to reconsider the criteria for interrupting or discontinuing Atezo + Bev, as in the protocol of the IMbrave150 clinical trial [2]. Although discontinuing Bev might help to resolve proteinuria in patients treated with Atezo + Bev, it may have a negative impact on prognosis. Furthermore, since this strategy is intended to preserve renal function, the lack of a correlation between the changes in proteinuria and decline in renal function may suggest that this approach is not always necessary. In fact, the Clinical Practice Guidelines for Management of Kidney Injury During Anticancer Drug Therapy 2022 [15,16] recommends that, if proteinuria occurs during VEGF inhibitor treatment, it is important to decide whether to interrupt or discontinue treatment after comprehensive evaluation of renal function and serum albumin levels, not just solely relying on the severity of proteinuria based on UPCR. Our findings support the clinical guidance and suggest that, even if proteinuria occurs, it is crucial to evaluate clinical symptoms such as hypoalbuminemia, edema, and ascites, and to consider continuing treatment based on a comprehensive clinical assessment.

### Limitations

Several limitations of this study should be acknowledged when interpreting the results. In particular, it was a single-center retrospective study with a relatively small sample size, which limited our ability to perform multivariable analyses to sufficiently adjust for multiple potential confounding factors. Furthermore, we did not consider the treatment duration in the analysis. Larger prospective multicenter studies with a longer follow-up are needed to validate these findings. Future studies should investigate the relationship between severe proteinuria and other AEs, including the impact of reserve liver function on albumin production.

## 5. Conclusions

In conclusion, the results of this study demonstrate that proteinuria during Atezo + Bev treatment is not necessarily associated with impaired renal function or clinically significant hypoalbuminemia in uHCC patients. Although Bev interruption criteria are based on the degree of proteinuria, our results suggest that proteinuria does not necessarily impair renal function. When deciding whether to discontinue Bev in patients who develop proteinuria, physicians should carefully evaluate the individual risk–benefit profiles, while considering renal function, serum albumin levels, and the severity of proteinuria. These findings may encourage physicians to continue Atezo + Bev without unnecessary treatment interruptions and to maximize its therapeutic benefits for patients. However, further validation through multicenter prospective studies and long-term follow-up is essential to confirm these observations and their clinical implications.

## Figures and Tables

**Table 1:** Patient characteristics.

Characteristic	Value	
*N*	100	
Age, years, median [range]	74.0 [41.0–89.0]	
Sex, male, *n* (%)	75 (75.0)	
ECOG PS 0, *n* (%)	88 (88.0)	
Etiology of HCC, *n* (%)		
HBV	21 (21.0)	
HCV	32 (32.0)	
Alcohol	16 (16.0)	
NAFLD & NASH	4 (4.0)	
BCLC stage, *n* (%)		
B	62 (62.0)	
C	38 (38.0)	
Child–Pugh class, *n* (%)		
A5	61 (61.0)	
A6	29 (29.0)	
B	10 (10.0)	
Treatment line, *n* (%)		
1st	51 (51.0)	
2nd/later	49 (49.0)	
Hypertension, yes, *n* (%)	60 (60.0)	
Diabetes, yes, *n* (%)	37 (37.0)	
Chronic kidney failure, yes, *n* (%)	6 (6.0)	
Cre, mg/dL		
Mean (SD)	0.87 (0.31)	
Median [range]	0.80 [0.42–2.46]	
UPCR, g/gCre		
Mean (SD)	0.52 (1.07)	
Median [range]	0.10 [0.02–5.68]	
Urine dipstick protein, *n* (%)		
−	55 (55.0)	
±	14 (14.0)	
1+	13 (13.0)	
2+	11 (11.0)	
≥3+	7 (7.0)	
eGFR, mL/min/1.73 m^2^		
Mean (SD)	67.8 (19.7)	
Median [range]	68.5 [21.0–113.0]	
BUN, mg/dL		
Mean (SD)	18.7 (6.9)	
Median [range]	17.5 [8.0–43.0]	
Serum albumin, g/dL		
Mean (SD)	3.7 (0.5)	
Median [range]	3.7 [2.5–4.7]	
History of TACE, yes, *n* (%)	50 (50.0)	

BCLC, Barcelona Clinic Liver Cancer; BUN, blood urea nitrogen; Cre, creatinine; ECOG PS, Eastern Cooperative Oncology Group performance status; eGFR, estimated glomerular filtration rate; HBV, hepatitis B virus; HCC, hepatocellular carcinoma; HCV, hepatitis C virus; NAFLD, non-alcoholic fatty liver disease; NASH, non-alcoholic steatohepatitis; SD, standard deviation; TACE, transcatheter arterial chemoembolization; UPCR, urine protein creatinine ratio.

**Table 2:** Univariate and multivariable logistic regression analyses of risk factors for renal function decline.

Variable	Univariate		Multivariable	
	OR [95% CI]	*p*	OR [95% CI]	*p*
Age (≥75 vs. <75 years)	1.09 [0.39, 3.03]	0.875	–	–
Sex (female vs. male)	2.60 [0.88, 7.70]	0.085	–	–
Hypertension (yes vs. no)	3.99 [1.19, 13.34]	0.025	2.70 [0.74, 9.83]	0.132
Diabetes (yes vs. no)	0.42 [0.12, 1.40]	0.156	–	–
BCLC stage (C vs. B)	0.52 [0.18, 1.55]	0.242	–	–
Child–Pugh class (B vs. A)	3.81 [0.70, 20.71]	0.121	–	–
Treatment line (2nd or later vs. 1st)	2.20 [0.77, 6.33]	0.142	–	–
UPCR (≥0.15 vs. <0.15) *	3.15 [0.75, 13.17]	0.117	–	–
eGFR (≤60 vs. >60 mL/min/1.73 m^2^)	5.50 [1.75, 17.26]	0.0035	3.72 [1.10, 12.61]	0.035
Serum albumin (≤3.5 vs. >3.5 g/dL)	1.18 [0.39, 3.58]	0.771	–	–
BUN (>17.5 vs. ≤17.5 mg/dL)	1.32 [0.47, 3.68]	0.602	–	–
History of TACE (yes vs. no)	3.60 [1.16, 11.19]	0.027	2.98 [0.90, 9.91]	0.075

*N* = 83. * Log_2_-transformed. Decreased renal function was defined as a decrease in eGFR to <50 mL/min/1.73 m^2^ during treatment with Atezo + Bev. Patients with eGFR < 50 mL/min/1.73 m^2^ at baseline were excluded from the analysis. OR, odds ratio.

**Table 3:** Univariate logistic regression analysis of risk factors for hypoalbuminemia (serum albumin < 3.0 g/dL).

Variable	Univariate	
	OR [95% CI]	*p*
Age (≥75 vs. <75 years)	0.57 [0.23, 1.44]	0.233
Sex (female vs. male)	1.14 [0.42, 3.08]	0.797
Hypertension (yes vs. no)	1.19 [0.47, 3.02]	0.707
Diabetes (yes vs. no)	0.73 [0.28, 1.94]	0.530
Chronic kidney failure (yes vs. no)	2.50 [0.47, 13.26]	0.282
BCLC stage (C vs. B)	1.49 [0.59, 3.74]	0.394
Treatment line (2nd or later vs. 1st)	1.09 [0.44, 2.69]	0.854
UPCR (≥0.15 vs. <0.15) *	3.31 [0.91, 12.01]	0.068
eGFR (≤60 vs. >60 mL/min/1.73 m^2^)	0.84 [0.32, 2.24]	0.731
Serum albumin (≤3.5 vs. >3.5 g/dL)	1.92 [0.70, 5.26]	0.203
BUN (>17.5 vs. ≤17.5 mg/dL)	1.26 [0.51, 3.11]	0.613
Cre (<1.5 vs. ≥1.5 mg/dL)	0.62 [0.10, 3.97]	0.618
History of TACE (yes vs. no)	1.35 [0.55, 3.32]	0.519

*N* = 90. Patients with serum albumin < 3.0 g/dL at baseline were excluded from the analysis. * Log_2_-transformed.

## Data Availability

The datasets generated during and/or analyzed during the current study are not publicly available for privacy or ethical reasons but are available from the corresponding author upon reasonable request.

## Supplementary Materials

The following supporting information can be downloaded at: https://www.mdpi.com/article/10.3390/cancers17233826/s1, Figure S1: Kaplan–Meier curves of overall survival in patients with normal renal function at baseline divided according to on-treatment renal function decline; Table S1: Characteristics of patients with normal renal function at baseline divided according to on-treatment renal function decline.

## Abbreviations

The following abbreviations are used in this manuscript:

AE	adverse event
ALBI	albumin-bilirubin
Atezo	atezolizumab
BCLC	Barcelona Clinic Liver Cancer
Bev	bevacizumab
BUN	blood urea nitrogen
CI	confidence interval
Cre	creatinine
ECOG PS	Eastern Cooperative Oncology Group performance status
EGD	esophagogastroduodenoscopy
eGFR	estimated glomerular filtration rate
HCC	hepatocellular carcinoma
HR	hazard ratio
ICI	immune checkpoint inhibitor
OR	odds ratio
OS	overall survival
PD-L1	programmed cell death ligand-1
TACE	transcatheter arterial chemoembolization
uHCC	unresectable hepatocellular carcinoma
UPCR	urine protein creatinine ratio
VEGF	vascular endothelial growth factor
