# Supplementary material for: No Correlation Between Proteinuria and Renal Function in Patients with Unresectable Hepatocellular Carcinoma Treated with Atezolizumab Plus Bevacizumab: ARISE Study
Source: Cancers (Basel). 2025 Nov 28;17(23):3826. doi: 10.3390/cancers17233826 (PMC12691049; doi:10.3390/cancers17233826)
Supplement: Supplementary file 1 [file cancers-17-03826-s001.zip › cancers-3685744-supplementary.pdf]

Journal: *Cancers*

Supplementary materials

No Correlation Between Proteinuria and Renal Function in Patients with Unresectable Hepatocellular Carcinoma Treated with Atezolizumab Plus Bevacizumab: ARISE Study

Authors: Kazuomi Ueshima, Naoshi Nishida, Satoru Hagiwara, Yasunori Minami, Hiroshi Ida, Masahiro Takita, Hirokazu Chishina, Masahiro Morita, Tomoko Aoki, Tetsutaro Hamano, Ryosuke Take, Chizuko Watanabe, Kohsuke Asoh, Ai Tanaka, Masatoshi Kudo

Corresponding author: Kazuomi Ueshima. E-mail: kaz-ues@med.kindai.ac.jp

**Contents**	**Page**
**Supplementary Table S1.** Characteristics of patients with normal renal function at baseline divided according to on-treatment renal function decline	2
**Supplementary Figure S1.** Kaplan–Meier curves of overall survival in patients with normal renal function at baseline divided according to on-treatment renal function decline	4

Supplementary Table S1. Characteristics of patients with normal renal function at baseline divided according to on-treatment renal function decline

**Characteristic**	**Non-decline group (*n* = 55)**	**Decline group (*n* = 10)**
Age, years, median [range]	72.0 [41.0–89.0]	74.5 [43.0–85.0]
Sex, male, *n* (%)	45 (81.8)	5 (50.0)
ECOG PS 0, *n* (%)	50 (90.9)	9 (90.0)
Etiology of HCC, *n* (%)		
HBV	13 (23.6)	4 (40.0)
HCV	15 (27.3)	2 (20.0)
Alcohol	10 (18.2)	3 (30.0)
NAFLD & NASH	2 (3.6)	0
BCLC stage, *n* (%)		
B	28 (50.9)	8 (80.0)
C	27 (49.1)	2 (20.0)
Child–Pugh class, *n* (%)		
A5	34 (61.8)	7 (70.0)
A6	19 (34.5)	1 (10.0)
B	2 (3.6)	2 (20.0)
Treatment line, *n* (%)		
1st	30 (54.5)	3 (30.0)
2nd/later	25 (45.5)	7 (70.0)
Hypertension, yes, *n* (%)	24 (43.6)	7 (70.0)
Diabetes, yes, *n* (%)	22 (40.0)	4 (40.0)
Chronic kidney failure, yes, *n* (%)	1 (1.8)	0
Cre, mg/dL		
Mean (SD)	0.72 (0.13)	0.74 (0.14)
Median [range]	0.73 [0.42–1.03]	0.71 [0.57–0.93]
UPCR, g/gCre		
Mean (SD)	0.31 (0.59)	0.62 (0.65)

Median [range]	0.08 [0.02–3.09]	0.35 [0.03–1.89]
Urine dipstick protein, *n* (%)		
−	34 (61.8)	4 (40.0)
±	7 (12.7)	1 (10.0)
1+	6 (10.9)	1 (10.0)
2+	6 (10.9)	4 (40.0)
≥3+	2 (3.6)	0
eGFR, mL/min/1.73 m^2		
Mean (SD)	80.7 (13.3)	71.2 (8.7)
Median [range]	78.0 [60.0–113.0]	71.0 [60.0–84.0]
BUN, mg/dL		
Mean (SD)	15.7 (4.5)	16.9 (3.8)
Median [range]	14.0 [8.0–26.0]	17.0 [12.0–23.0]
Serum albumin, g/dL		
Mean (SD)	3.7 (0.5)	3.6 (0.5)
Median [range]	3.8 [2.6–4.7]	3.7 [2.7–4.3]
History of TACE, yes, *n* (%)	24 (43.6)	6 (60.0)

Normal baseline renal function was defined as an eGFR ≥60 mL/min/1.73 m^2. Renal function decline was defined as an eGFR <50 mL/min/1.73 m^2

BCLC, Barcelona Clinic Liver Cancer; BUN, blood urea nitrogen; Cre, creatinine; ECOG PS, Eastern Cooperative Oncology Group performance status; eGFR, estimated glomerular filtration rate; HBV, hepatitis B virus; HCC, hepatocellular carcinoma; HCV, hepatitis C virus; NAFLD, non-alcoholic fatty liver disease; NASH, non-alcoholic steatohepatitis; SD, standard deviation; TACE, transcatheter arterial chemoembolization; UPCR, urine protein creatinine ratio

Supplementary Figure S1. Kaplan–Meier curves of overall survival in patients with normal renal function at baseline divided according to on-treatment renal function decline

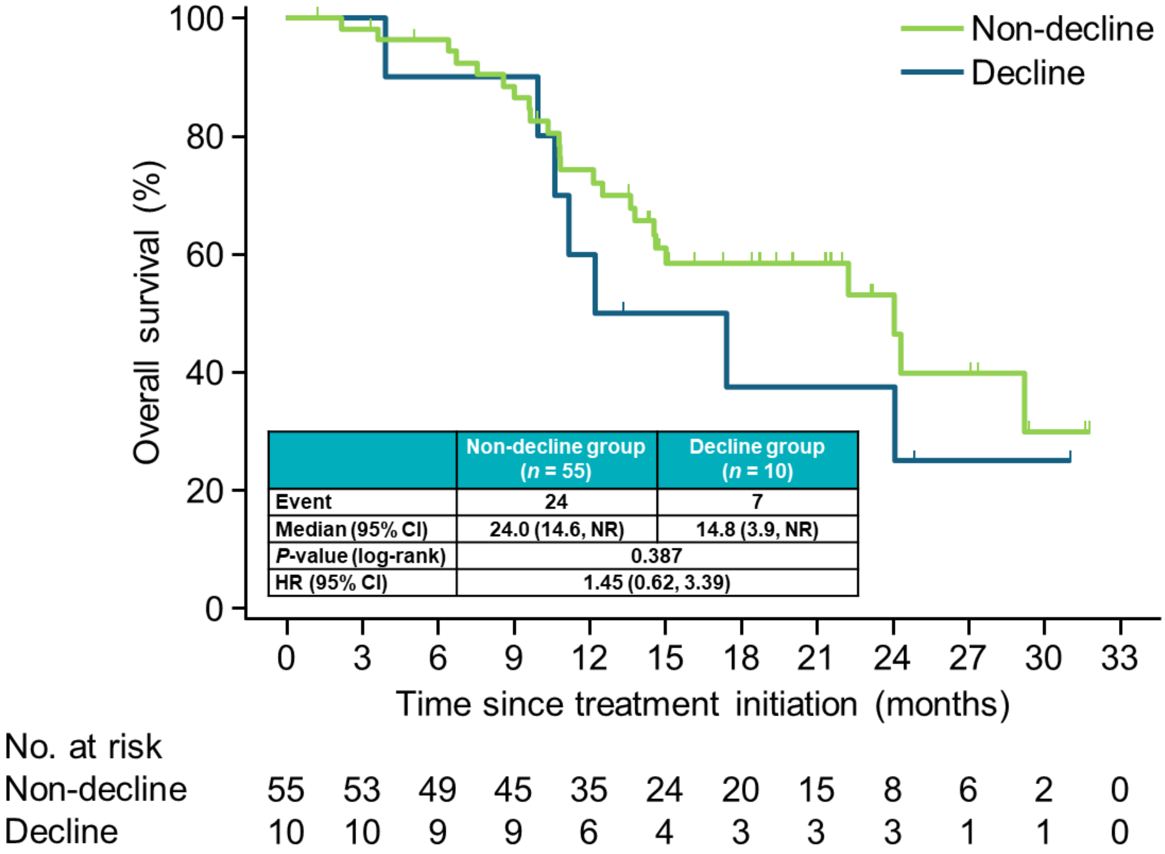

	Non-decline group (n = 55)	Decline group (n = 10)
Event	24	7
Median (95% CI)	24.0 (14.6, NR)	14.8 (3.9, NR)
P-value (log-rank)	0.387	
HR (95% CI)	1.45 (0.62, 3.39)	

Normal baseline renal function was defined as an eGFR ≥60 mL/min/1.73 m². Renal function decline was defined as an eGFR <50 mL/min/1.73 m²

CI, confidence interval; eGFR, estimated glomerular filtration rate; HR, hazard ratio; NR, not reached
